# Printed n- and p-Channel Transistors using Silicon Nanoribbons Enduring Electrical, Thermal, and Mechanical Stress

**DOI:** 10.1021/acsami.2c20569

**Published:** 2023-02-12

**Authors:** João Neto, Abhishek Singh Dahiya, Ayoub Zumeit, Adamos Christou, Sihang Ma, Ravinder Dahiya

**Affiliations:** † James Watt School of Engineering, University of Glasgow, Glasgow G12 8QQ, United Kingdom; ‡ Bendable Electronics and Sustainable Technologies (BEST) Group, Electrical and Computer Engineering Department, Northeastern University, Boston, Massachusetts 02115, United States

**Keywords:** printed electronics, flexible electronics, transistors, silicon nanoribbons, endurance

## Abstract

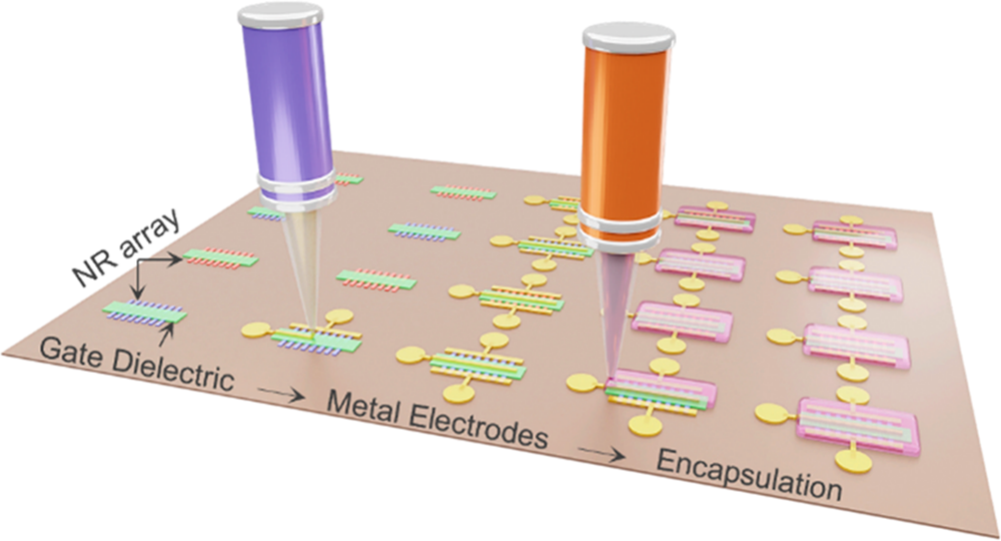

Printing technologies are changing the face of electronics with features such as resource-efficiency, low-cost, and novel form factors. While significant advances have been made in terms of organic electronics, the high-performance and stable transistors by printing, and their large-scale integration leading to fast integrated circuits remains a major challenge. This is because of the difficulties to print high-mobility semiconducting materials and the lack of high-resolution printing techniques. Herein, we present silicon based printed n- and p-channel transistors to demonstrate the possibility of developing high-performance complementary metal–oxide–semiconductor (CMOS) computing architecture. The direct roll transfer printing is used here for deterministic assembly of high-mobility single crystal silicon nanoribbons arrays on a flexible polyimide substrate. This is followed by high-resolution electrohydrodynamic printing to define source/drain/gate electrodes and to encapsulate, thus leading to printed devices. The printed transistors show effective peak mobilities of 15 cm^2^/(V s) (n-channel) and 5 cm^2^/(V s) (p-channel) at low 1 V drain bias. Furthermore, the effect of electrical, mechanical, and thermal stress on the performance and stability of the encapsulated transistors is investigated. The transistors showed stable transfer characteristics even after: (i) continuous 4000 transfer cycles, (ii) excruciating 10000 bending cycles at different bending radii (40, 25, and 15 mm), and (iii) between 15 and 60 °C temperatures.

## 1 Introduction

Over the last 60 years, the miniaturization of integrated circuits (ICs) has revolutionized computing and communication capabilities. The phenomenal progress achieved through large scale integration of miniaturized high-speed devices has enabled fast digital technologies touching life in almost all traditional sectors today (e.g., health, aerospace, manufacturing, retail displays, robotics, wearable systems, etc.).^1−9^ Yet, as revolutionary as this miniaturization trend has been, in its current form, the production processes it follows are inherently and unavoidably wasteful. For instance, IC fabrications rely almost entirely on subtractive manufacturing methods (sequence of photolithographic and chemical processing steps), leading to large material wastages, and high levels of anions and organic pollutants.^10−12^ Such methods are not suitable for large area flexible electronics that can bend, flex, and twist. Even if advances with silicon-based technologies continue to be made, there is need for alternative technology offering resource-efficient and environment friendly routes for manufacturing electronics without losing its transformative power.

Printed electronics is rapidly changing the way electronics is manufactured and used.^13−16^ With resource-efficient and low-cost fabrication of electronics over large areas (larger than the commercially available wafers) and flexible and stretchable substrates, the printed electronics is advancing several applications.^13,17−22^ Efficient use of various materials also makes printing attractive in terms of lower electronic waste (e-waste) and better environment friendliness.^11−13,23−25^ As a result, the printing technologies have been explored for devices such as artificial thermoreceptors,^26^ touch sensors,^27^ synaptic transistors,^28^ energy harvesters,^29−31^ radio frequency identification (RFID) tags,^32^ and interconnects,^33^ etc. needed in conformable and interactive electronic systems.^26,28,34^ However, the nonavailability of high-performance (i.e., low power, fast speed) processing units has restricted the utility of printed electronics to low-end applications.

Transistors form the basic building blocks for any processing unit, and several research groups are working toward the development of high-performance printed transistors. For example, significant advances have been made by depositing thin films of organic and metal oxide materials and employing variety of printers such as gravure,^17,35^ inkjet,^18^ etc. The thin-film transistors can be lightweight, flexible, and conformal and they can be manufactured on large substrates at low-cost per unit area.^36−40^ However, high operating voltages (up to 50 V), low mobility (<1 cm^2^/(V s)), poor current injection, and low integration density remain challenges for organic thin-film transistors (OTFTs). The current benchmark is a flexible microprocessor based on indium–gallium–zinc oxide (IGZO) TFTs developed using conventional microfabrication processes.^37^ However, due to availability of n-channel transistors only, the circuits based on this technology are more complicated and power-hungry.^41,42^ Further, the obtained n-channel device mobility (<10 cm^2^/(V s)) is insufficient for high-speed applications. Transfer printing of ultrathinned chips (UTCs) over flexible substrates can provide short-term practical solutions to satisfy the need of high performance, but the technology has its own limitations such as high fabrication cost, and limited flexibility.^33,43,44^ To overcome these bottlenecks, printing of high carrier mobility electronic layers and the optimization of high-resolution printing technologies is required.

Driven by these challenges, our approach is to develop printed transistors on flexible substrates employing high intrinsic mobility Si nanoribbons (NRs) and a high-resolution drop-on-demand (DoD) printer. To integrate Si NR arrays, single-step direct roll transfer printing method is used which has shown potential for large area electronics.^45−47^ This newly developed printing technique avoids the use of elastomeric stamps (typically employed in transfer printing) and thus, reduces the number of fabrication steps. Using the direct roll transfer approach, arrays of both n- and p-channel Si NRs are printed over flexible polyimide (PI) substrates. Next, high-resolution DoD electrohydrodynamic (EHD) printing was utilized to print Au source/drain/gate (S/D/G) metal electrodes. EHD offers excellent possibilities such as high-resolution patterning (≈1 μm) needed to reduce the channel length of transistors, substrate independent patterning, and the potential for low-cost operation.^48−50^ Moreover, it shows a better compatibility with automated control, potentially leading to resource-efficient “digital manufacturing”. The developed transistors show moderately high effective mobilities (*n*_e_ = 15; *n*_p_ = 5 cm^2^/(V s)) at low drain bias (≈1 V). Furthermore, EHD was employed to encapsulate the printed transistors with nanosilica/epoxy (NS/epoxy) dielectric material. The encapsulated transistors showed stable response even after excruciating 10000 bending cycles at different bending radii (40, 25, 15, and 5 mm) and at both low (15 °C) and high (60 °C) temperatures. Through these proof-of-concept devices, this work provides new resource-efficient route for printed electronics and flexible low-power complementary metal–oxide–semiconductor (CMOS) logic circuits.

## 2 Result and Discussions

### 2.1 Fabrication of Printed n- and p-Channel Transistors

**Figure 1:**
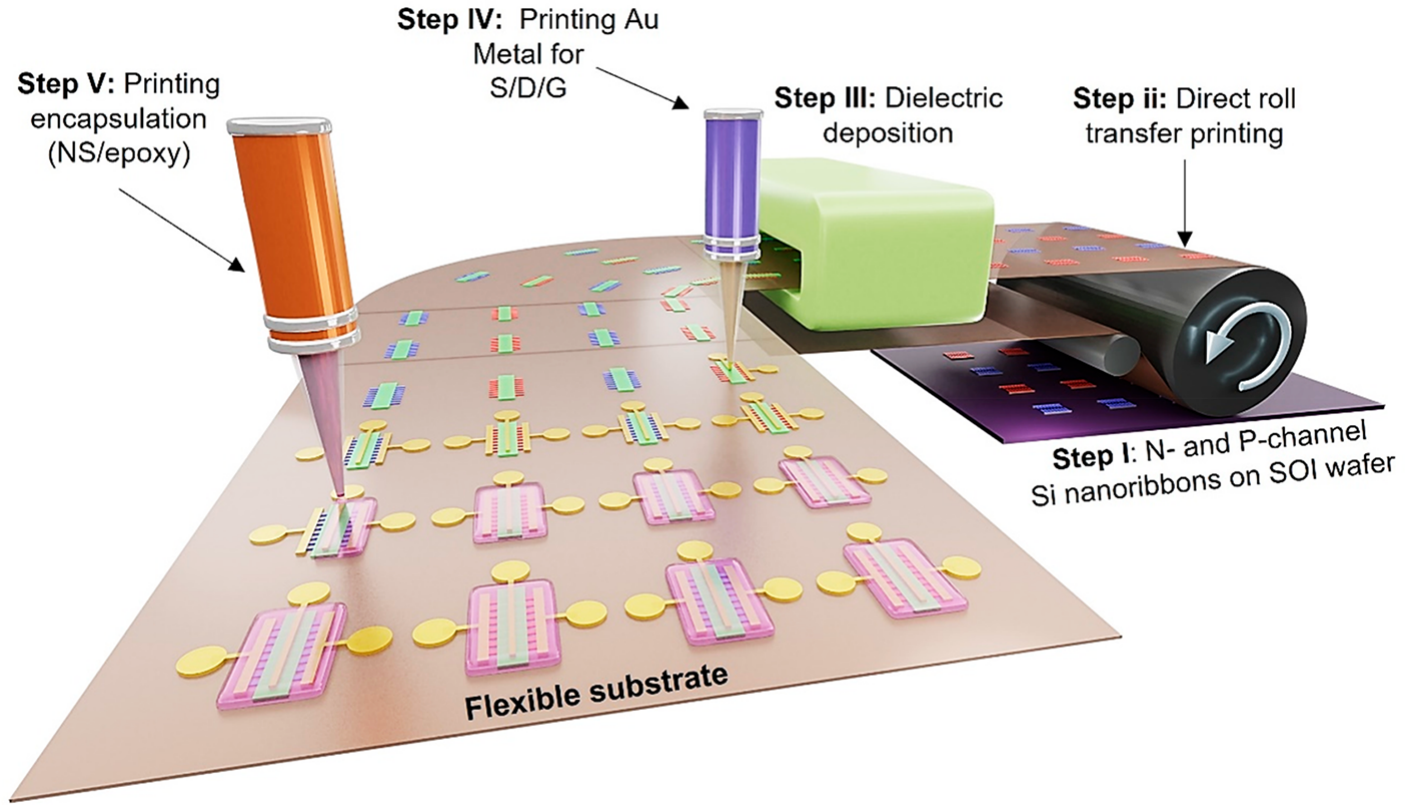
Schematic of the fabrication process steps for printed n- and p-channel transistors. (i) n- and p-type NRs fabricated and anchored to the donor wafer. (ii) Direct roll transfer printing of NRs on a semicured flexible polyimide substrate. (iii) PECVD deposition of SiN_*x*_ at room temperature over the transferred NRs. (iv) EHD printing of Au metallic ink over the drain/source and gate regions. (v) EHD patterned printing of NS/Epoxy encapsulant over the fabricated transistors.

Figure 1 schematically shows the developed fabrication process flow of printed transistors on flexible substrates. The details of the materials and process conditions are given in the “Methods” section. The process starts with the fabrication of intrinsically high mobility Si NRs arrays on a rigid silicon-on-insulator (SOI) wafer (step i, Figure 1). The NR’s dimensions were defined using conventional nanofabrication processes including photolithography and etching, as described in our previous works.^59^ The selective doping step was performed using spin-on-dopant (SoD) technique on NRs arrays to realize n- and p-channel devices (see methods for details). The horizontally aligned, suspended arrays of doped NRs over SOI source wafers are then transfer printed on to flexible receiver substrates. There are different routes to execute transfer printing of NRs. In the present work, we have employed single-step ‘direct roll transfer printing’ method to transfer n- and p-type doped NRs (step ii, Figure 1).^46^ The printing technique has shown potential for large area electronics by avoiding the use of elastomeric stamps. The use of such stamps during transfer printing methods generally leads to low transfer yield, and registration issues. The direct roll transfer printing employed here not only reduces the number of fabrication steps with respect to the conventional transfer printing approach but also offers opportunity for roll-to-roll (R2R) manufacturing. In this method, suspended arrays of NRs over the SOI substrate are brought into direct physical contact with the spun semicured PI layer over the receiver substrate. The semicured PI layer helps to attain a high transfer yield. The printed Si NR arrays were then processed to realize flexible transistors. As a high-quality dielectric layer, we deposited silicon nitride (SiN_*x*_) at room temperature (RT) using plasma-enhanced chemical vapor deposition (PECVD). Using photolithography and dry etching we then opened the regions for metal contacts on NRs (step iii, Figure 1). The doping was performed to (i) have p- and n-channel devices using single source SOI wafer, and (ii) achieve low-contact resistance metal semiconductor (MS) contacts to nanostructures. The diffusive gold (Au) metal is then printed by employing high-resolution EHD printer (step iv, Figure 1) to define S/D/G electrodes. An optimized Ar plasma treatment was performed before printing Au metal to remove any organic residues and to improve the wettability of the Au ink with the substrate. Further, we believe that the plasma treatment can help to remove any insulating layer existing on the surface of NRs and thus to achieve high quality interface between Au and heavily doped silicon. It is to note that the plasma unit is installed within the EHD printing chamber. The Au ink was cured at 250 °C for 2h to improve the conductance of the printed structures. Finally, the transistors were encapsulated by printing NS/epoxy layer using EHD printer (step v, Figure 1). The optimization of the EHD printing for Au and NS/epoxy inks is described elsewhere^26^ and details are provided in “Methods” section.

### 2.2 Electrical Characterization of Printed n- and p-Channel Transistors

**Figure 2:**
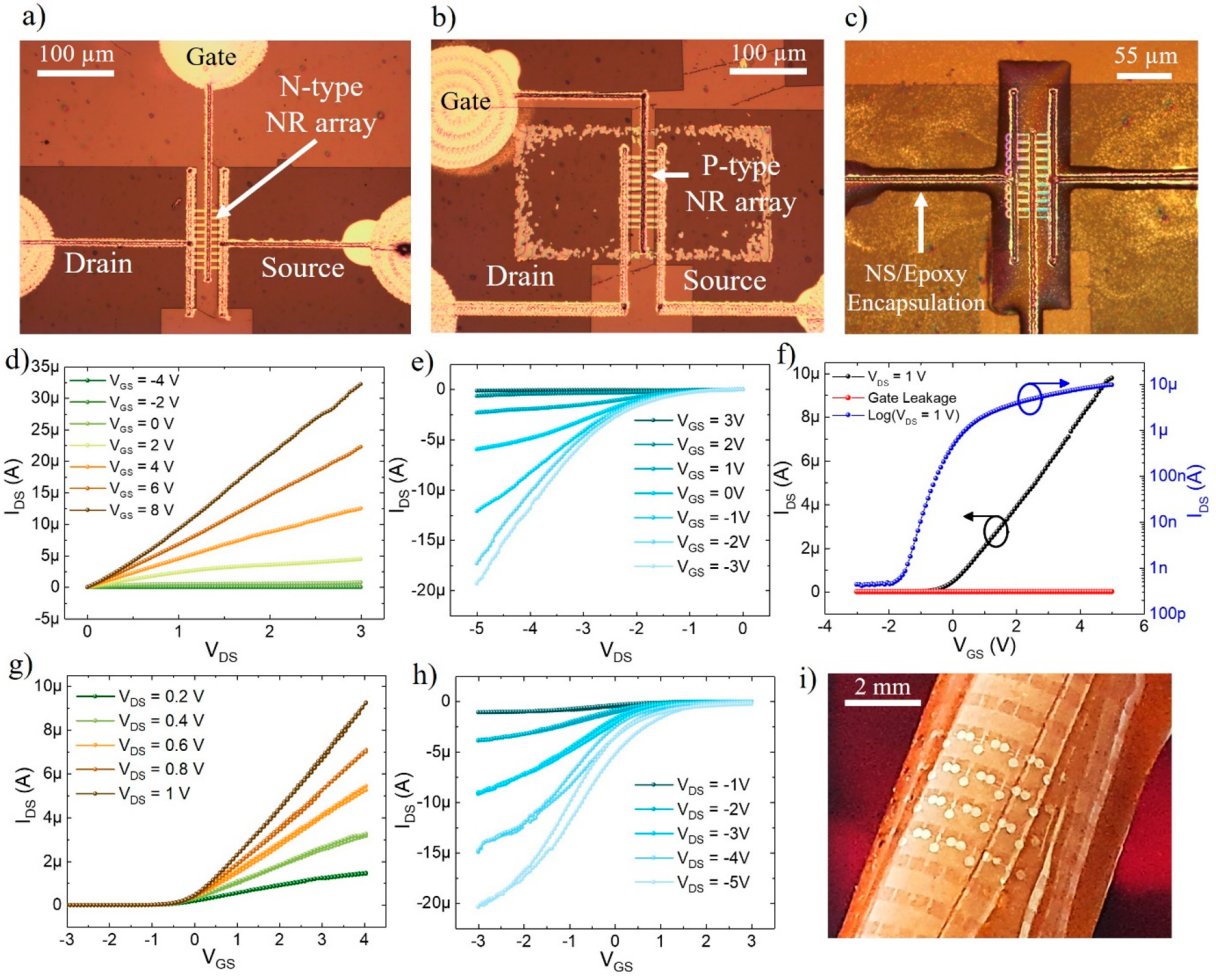
Electrical characteristics of printed n- and p-channel transistors. (a–c) Optical micrograph of the printed transistor (scale bar, 100 μm). (d) Output characteristics of n-channel printed transistor. (e) Output characteristics of p-channel printed transistor. (f) Transfer characteristics with *V*_DS_ = 1 V in logarithmic and linear scales. The graph also shows the gate leakage current. (g) Transfer characteristics of n-channel transistor with *V*_DS_ varying from 0.2 to 1 V with the step of 0.2 V. (h) Transfer characteristics of p-channel transistor with *V*_DS_ varying from −1 V to −5 V with the step of −1 V. (i) Optical picture of printed transistors while placed on a cylindrical tube.

Figure 2 depicts the electrical characterization results and optical image of a typically fabricated and best performing n- and p-channel printed transistor. The statistical data from five different devices for each n- and p-channel is shown in Figures S1 and S2, respectively. The Figure 2a–c show optical images of fabricated n-channel and p-channel printed transistors. The typical channel length (*L*_ch_) and width (*W*_ch_) of the fabricated transistors (both n- and p-channel) are ∼5 and ∼45 μm (9 NRs of 5 μm wide), respectively. Figure 2c represents optical image of a n-channel encapsulated device. Representative output curves for n- and p-channel transistors are shown in Figure 2d,e, respectively. The data evidently shows good linearity in the output curve for n-channel devices at low drain bias, indicating that the ohmic nature of source and drain contacts. However, presence of crowding behavior in the output curve at low drain bias (n-channel) suggests the presence of an injection barrier at the MS contacts which is comparatively higher than the conventional devices (where metal is defined using evaporation method under high vacuum conditions).^46^ The presence of extra resistances in series degrades the transistor performance.

The critical transistor performance metrics include on-state current (*I*_on_), off-state current (*I*_off_), current on/off ratio (*I*_on_/*I*_off_), transconductance (*g*_m_), effective device mobility (μ_*eff*_), and subthreshold slope (S–S).^60^ These parameters are important as they help to benchmark the transistors developed using low-dimensional materials and novel fabrication technique. Along with the performance metrics, transistor structural parameters that influence device performance include *L*_ch_, *W*_ch_, gate insulator thickness and permittivity, contact metal types (ohmic/Schottky), the thickness of the channel material, contact resistance (*R*_c_) etc. The performance metrics were extracted using the transfer characteristics (*I*_DS_–*V*_GS_) performed with *V*_DS_ = 1 V for a transistor with *L*_ch_/*W*_ch_ ratio ≈5/45, channel thickness ≈70 nm, and gate insulator thickness and permittivity of 100 nm and ∼7, respectively. Figure 2f shows the plot in logarithmic and linear scales. The measured devices showed typical *I*_on_ (∼10 μA)/*I*_off_ (<1 nA) current ratio of >10^4^ suggesting an excellent gate-channel control. The extracted S–S from the logarithm transfer plot is 0.4 V/decade. Next, threshold voltage (*V*_T_) was extracted using the linear extrapolation method. For this, the linear extrapolation of *I*_DS_–*V*_GS_ graph, intercepting the *I*_d_ = 0 at *x*-axis (*V*_GS_) gives the *V*_T_ value (−0.2 V). This was followed by the calculation of transconductance (*g*_m_), as per eq 1 below:
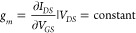
1

The calculated peak *g*_m_ is ∼2.5 μS at *V*_DS_ = 1 V. Next, the field-effect (effective) device mobility was extracted using the conventional MOSFET model in the linear regime:^46,47,61^
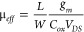
2The extracted effective mobility was found to be ∼15 cm^2^/(V s). Depending on the structural device parameters such as *R*_c_ values, μ_*eff*_ can be underestimated or sometimes overestimated relative to the drift mobility of the channel material.^62^ For instance, substantial *R*_c_ which is typical to low-dimensional materials–metal interfaces pose major challenge in understanding intrinsic charge transport properties. In past, few methods have been proposed to make μ_eff_ less dependent on these factors but none of them is sufficiently general enough to be widely adopted. Further, it is hard to extract the *R*_c_ values in the present case as widely adopted transfer length method (TLM) requires a series of transistors with different channel lengths having consistent contact and gating configurations. It is challenging to have consistent MS contact configuration to extract near-ideal *R*_c_ for printed transistors. Likewise, in this work, we have used the often-used approach to extract field effect mobility (eq 1) and compared it with the state-of-the-art printed transistors. Although the obtained mobility value compares well with the state-of-the-art n-channel printed transistors (Table 1), it is 2 orders of magnitude lower than its intrinsic value (μ_n_ = 1500 cm^2^/(V s)) and ∼40 times lower than flexible Si transistors (≈650 cm^2^/(V s)) realized using conventional microfabrication approach.^46^ This could be attributed to the high *R*_c_ of the printed transistors compared to the conventionally made devices where the metal is deposited under ultrahigh vacuum conditions.

**Table 1:** Comparison of the Printed n- and p-Channel NR Transistors with Other State-of-the-Art Printed Transistors^a^

channel material (printing method)	dielectric (deposition method)	S/D contacts (printing method)	mobility (cm^2^/(V s))	current on/off ratio	flexible	bending cycles (radius)	ref
SWCNT (inkjet)	HfO_2_ (ALD)	Au (evaporation)	p-type: 10–30	10^6^	no	N/A	(51)
			n-type: 10–30				
SWCNT (inkjet)	HfO_2_ (ALD)	Au (evaporation)	17.6–37.7	10^4^–10^7^	no	N/A	(52)
SWCNT (aerosol)	SiO_2_	CNT (aerosol)	7	10^6^	no	N/A	(53)
SWCNT (inkjet)	PMMA (spin-coated)	GO (inkjet)	8	10^4^	yes	N/A	(54)
IrGO (rod)	SiO_2_	GO (inkjet)	4.37		yes	N/A	(55)
MoS_2_ (inkjet)	Electrolyte (inkjet)	Cr/Au (evaporation)		10^6^	no	N/A	(56)
MoS_2_ (spin-coating)	SiO_2_/Si (deposition)	Pt	7–11	10^6^	no	N/A	(57)
SnO_2_ (inkjet)	Al_2_O_3_ (inkjet precursor)	ITO (inkjet precursor)	13.3	10^5^	yes	1000 (5 mm)	(58)
Si NR (transfer printing)	SiN_*x*_ (PECVD)	Au (inkjet)	n-type: 15	10^4^	yes	10k (45–25–15 mm)	this work
			p-type: 5				

a ALD: atomic layer deposition; CNT: carbon nanotubes; GO: graphene oxide; ITO: indium tin oxide; Pt: platinum; and N/A: data not available.

Further, transfer characteristics with *V*_DS_ varying from 0.2 to 1 V with the step of 0.2 V is performed (Figure 2g). As shown in this figure, there is a constant increase in the output current with increase of *V*_DS_ while no change in threshold voltage was observed. Like the n-channel devices, the p-channel transistors were electrically characterized under similar condition. Representative output curves (Figure 2e) clearly show nonlinearity in the output curves at the low drain bias, indicating existence of a larger injection barrier as compared to n-channel transistors. Consequently, as explained above the effect of *R*_c_ on device mobility, extracted mobility using the transfer scan (Figure 2h) is lower (5 cm^2^/(V s)) than the n-channel transistors. For the n-channel devices, almost no hysteresis was observed (Figure 2g). Like n-channel devices, negligible hysteresis was observed for p-channel devices at lower *V*_DS_ confirming the good quality of the gate dielectric (Figure 2h), but at higher *V*_DS_ (−5 *V*_DS_), the hysteresis is ∼0.4 V which is still lower than the reported nanoscale transistors.^63^ There are various factors related to the hysteresis width such as device geometry (dielectric thickness, etc.), environmental conditions (temperature and humidity), and measurement parameters (gate sweeping rate, etc.).^64^ The observed small hysteresis in p-channel devices could be possibly because of the charges injected onto trap sites (surface traps, interface traps and dielectric traps) at higher electrical field. The extracted performance metrics for both n- and p-channel transistors are shown in Table 2.

**Table 2:** Summary of the Key Performance Metrics for the Fabricated Flexible Printed n- and p-Channel Transistor Device^a^

channel type	*V*_DS_	*I*_on_ (A)	*I*_off_ (A)	*I*_on/off_ ratio	hysteresis (Δ*V*)	μ_eff_ (cm^2^/V s)	S–S (V/dec)	*V*_T_ (V)
n-type	1 V	9 μ	400p	10^4^	0.15	15	0.4	–0.2
p-type	–4 V	18 μ	200n	10^2^	0.2	5	2 V	0.8

a The data is shown for the best obtained device for each type.

It is to note that the fabricated transistors (both n- and p-channel) showed some device-to-device performance variations including field-effect mobility, S–S etc. We have characterized five devices of each n- and p-channel printed NRFET, and a statistical device variation is included in Figures S1 (n-channel) and S2 (p-channel) and summarized in table S1. To improve the uniformity of device response from single batch or from different batches, many aspects need attention. First, the uniform, and controlled doping of the donor substrate need to be ensured. The doping step in this work is performed using the spin-on-dopant approach under ambient pressure which was not an ideal approach and could lead to nonuniformity in the devices. Second, highly controlled NR printing approach is needed to have uniform electronic layer. The direct roll transfer printing approach has shown very good transfer yield of ∼95% but there is still room for improvements. Last, the performance of printed NRFETs is influenced by the quality of metal–semiconductor contact interface and the dielectric/semiconductor interface. Because of the low dimensionality of NRs and adopted printed route to realize metal contacts, it is challenging to realize high quality contacts primarily due to the electrostatics involved at nanocontact interfaces. Currently, we are working on these aspects to further reduce the batch-to-batch performance variations of devices and will present the advances in our future works.

To evaluate the potential of the fabricated printed transistors in the development of high-speed circuits, the theoretical cutoff frequency *f*_*T*_ is extracted using the following equation:^65^
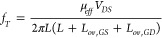
3where *L_ov,GS_* is the parasitic gate-to-source overlap and *L_ov,GD_* is the parasitic gate-to-drain overlap. Neglecting the parasitic capacitances, the *f*_*T*_ of n-channel transient transistor with a channel length of 5 μm and mobility of 15 cm^2^/(V s) is estimated to be more than 19 MHz, while for p-channel transistor it is 9.5 MHz which can be further enhanced by reducing the channel length and contact resistance in future optimized design. Nevertheless, it is still higher than the state-of-the-art printed transistor devices based on organics and metal oxides.^37^

### 2.3 Reliability Studies of Printed Transistors

#### 2.3.1 Electrical Bias Stability

**Figure 3:**
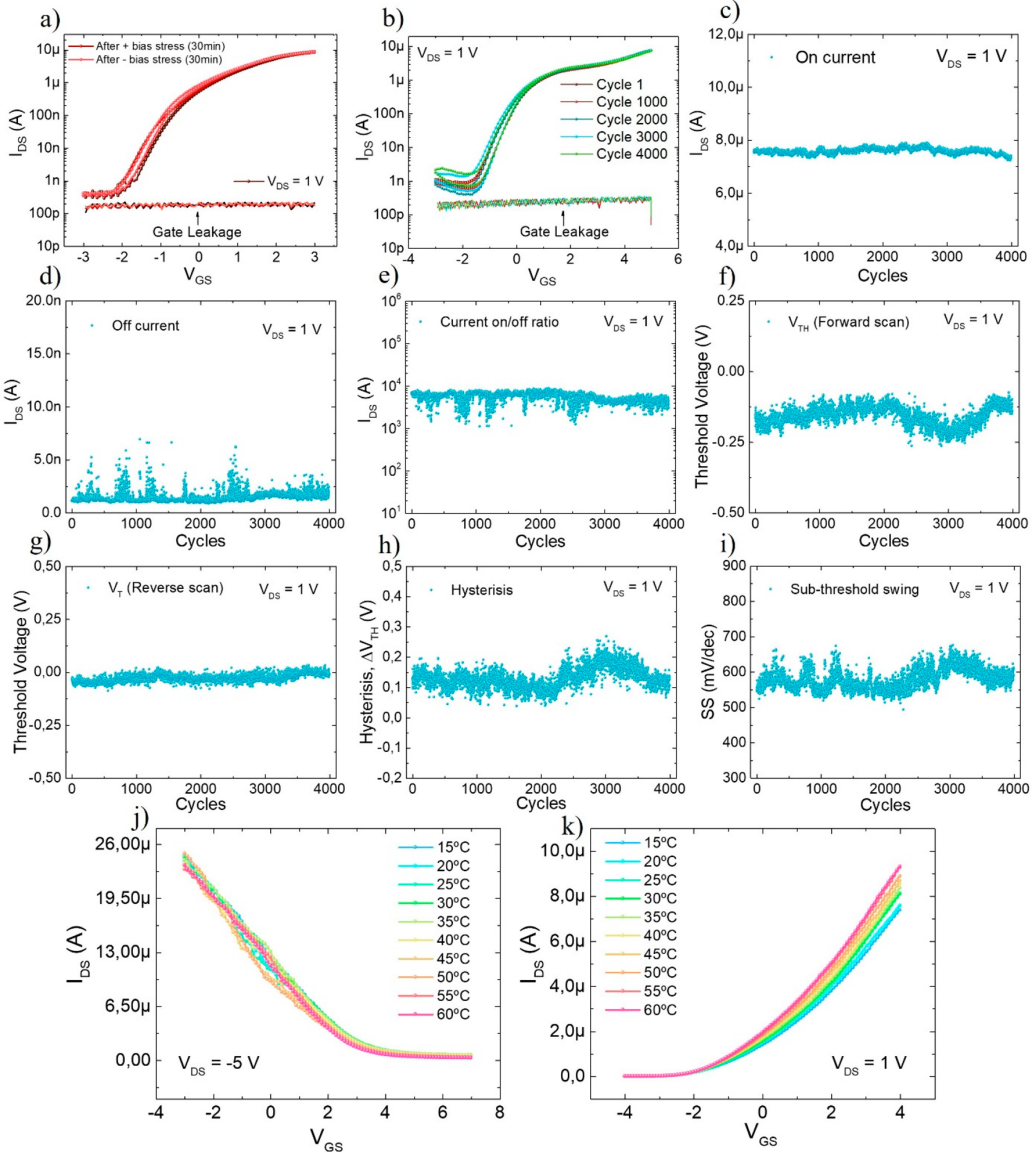
Electrical bias and thermal stress performance evaluation of printed transistors. a) Transfer curves with gate leakage after positive and negative gate bias stress of 30 min. b) 4000 transfer scan measurements with gate leakage at *V*_DS_ = 1 V. (c–i) Extracted key transistor parameters from the cyclic transfer scan regarding the device performance stability: c) on-current, d) off-current, e) current on/off ratio, f) threshold voltage (forward scan), g) threshold voltage (reverse scan), h) hysteresis, and i) subthreshold swing. j, k) Transfer scans with a temperature range of 15–60 °C with 5 °C step for both p- and n-type devices.

Stable transistor operation is required for any practical application. The stability of device operation could be affected during continuous gate electrical bias stress, temperature change, mechanical loadings, etc.^60,66^ Further, exposure of active channel to ambient atmosphere could lead to interactions with water and oxygen molecules, and thus to the charge trapping at the surface states, which eventually lead to surface band bending and shift in the threshold voltage, on-current, etc.^28^ These factors are more dominant in nanoscale transistors due to the high surface to volume ratio of nanomaterials. These drawbacks could be carefully utilized to develop novel devices such as printed synaptic transistor-based electronic skin allowing robots to feel and learn;^28^ they should be minimal when it comes to ICs. Therefore, we encapsulated the printed transistors using NS/epoxy of thickness ∼2–3 μm to reduce the influence of environment on NR channel (Figure 2c). To evaluate the stability of printed transistors, we performed the following electrical tests: (i) gate bias stress, (ii) continuous device operation up to 4000 cycles, (iii) transfer scans at wide range of temperatures, (iv) and transfer scans to evaluate the degradation of devices performance after applying stringent mechanical bending loadings. The results from the first three stability tests are shown in Figure 3. First, positive bias stress (PBS), and negative bias stress (NBS) stability tests were performed (Figure 3a). The transfer scan was performed under no stress condition by sweeping the *V*_GS_ from −3 to +3 V at a fixed 1 *V*_DS_. Both for PBS and NBS test, transfer scans were performed immediately after applying the bias for 30 min. As shown in Figure 3a, the printed transistors under NBS and PBS exhibits negligible drift of the transfer curves, and threshold voltage shifts (Δ*V*_T_) after 30 min stress time. The presented data hints presence of a high-quality of deposited gate dielectric, gate dielectric (SiN_*x*_)-semiconductor (Si NR) interface of the fabricated transistor and no influence of ambient condition on the performance degradation. This is critical because poor interface quality could lead to *V*_T_ shift and thus, changes in the *I*_on_ of the device.^60^ In our previous works we have confirmed the high electrical quality of the RT deposited SiN_*x*_ as gate dielectric.^59^ However, unlike the previous case where conventional manufacturing process was adopted, in the present case all printed route is employed to also deposit the metal tracks which can bring additional challenges. For instance, the RT deposition could lead to a porous dielectric layer and thus, there is a possibility of the liquid printed Au ink, over the deposited SiN_*x*_ film, to flow through the pores during the curing process and short circuit the channel with the printed top gate. If not short-circuit, then such infiltration of Au ink inside the dielectric could also lower the effective thickness of the dielectric leading to lower breakdown voltages.^58^ To have a good quality dielectric layer, we performed the annealing step at 250 °C for 2h in a conventional oven before printing the top-gate electrode. The annealing time and temperature were chosen in accordance with the curing conditions of printed Au ink. The excellent gate coupling and gate bias stability seen in the presented results hints that the deposited room temperature SiN_*x*_ after thermal annealing is dense enough to prevent the ink from infiltrating.

To further confirm the high stability, we have carried out electric bias stress where the transfer scans were performed continuously up to 4000 cycles (>24 h). The *I*_DS_ was monitored by sweeping the *V*_GS_ from −5 to +5 V (forward voltage sweep) and from +5 to −5 V (reverse voltage sweep) at a constant *V*_DS_ = 1 V (classified as one stress cycle). This is because the presence of any hysteresis due to charge trapping can further provide information on the quality of interfaces and dielectric. Figure 3b shows five transfer scans corresponding to cycles 1, 1000, 2000, 3000, and 4000. From this data it appears that the device remained stable when they were operated for more than a day. To have further insights into all 4000 cycles, the performed transfer scans were used to extract the key transistor performance metrics, namely, *I*_on_, *I*_off_, *I*_on/off_ ratio, hysteresis (from the *V*_T_ value of forward and reverse scan), and S–S. Throughout the 4000 electrical cycles, the *I*_on_ (Figure 3c) showed stable values (change of ∼4%); however, few cycles have shown significant increase in *I*_off_ by 100% (Figure 3d) leading to a decrease in *I*_on_/*I*_off_ (Figure 3e) ratio to ≈10^3^ for those particular cycles. However, on average, only 28% decrease in the *I*_on_/*I*_off_ current ratio was observed.

Next, hysteresis (Figure 3h) is calculated based on the difference in *V*_T_ value for forward (Figure 3f) and reverse (Figure 3g) transfer scans. For forward scan there is small *V*_T_ shift (±0.1 V) with the stress time whereas reverse scans showed negligible *V*_T_ shift. This value is lower, when compared with the reported transistors-based metal oxides.^60,67^ Using the *V*_T_ values, a maximum of ∼25% change in hysteresis values was obtained. Finally, S–S values were obtained from the subthreshold region of the transfer curve and plotted in Figure 3i. The data shows ∼8% change in S–S value after 24 h of continuous operation.

In addition to the electrical stress, nanoscale transistors are known to be susceptible to thermal stress.^68,69^ To evaluate the performance degradation while varying the ambient temperature, we performed transfer scans in forward direction (−4 to +4 *V*_GS_) for n-channel (Figure 3j) and +7 to −3 *V*_GS_ for p-channel transistors (Figure 3k) at different temperatures (15 to 60 °C with a step of 5 °C). The *I*_on_ of the device (n-channel) shows slight but consistent increase in values with temperature increase. Thanks to the PI substrate, which has a very wide operation temperature range, the stable electrical performance was observed. The Si NRs as semiconductor channel and SiN_*x*_ as dielectric can also endure the experimented temperature range. The slight increase in the on-current of the n-channel device with increasing temperatures is owing to the presence of charge injection barrier at the MS interface. At higher temperature, the charge carriers acquire more kinetic energy to cross over the present barrier at the MS interface.^68,69^ Interestingly, despite observed increase in the on-current, there is no change in *V*_T_ values, which further confirms the high quality of dielectric and semiconductor-dielectric interfaces. For p-channel transistors, there is no change in *I*_on_ as well as *V*_T_.

**Figure 4:**
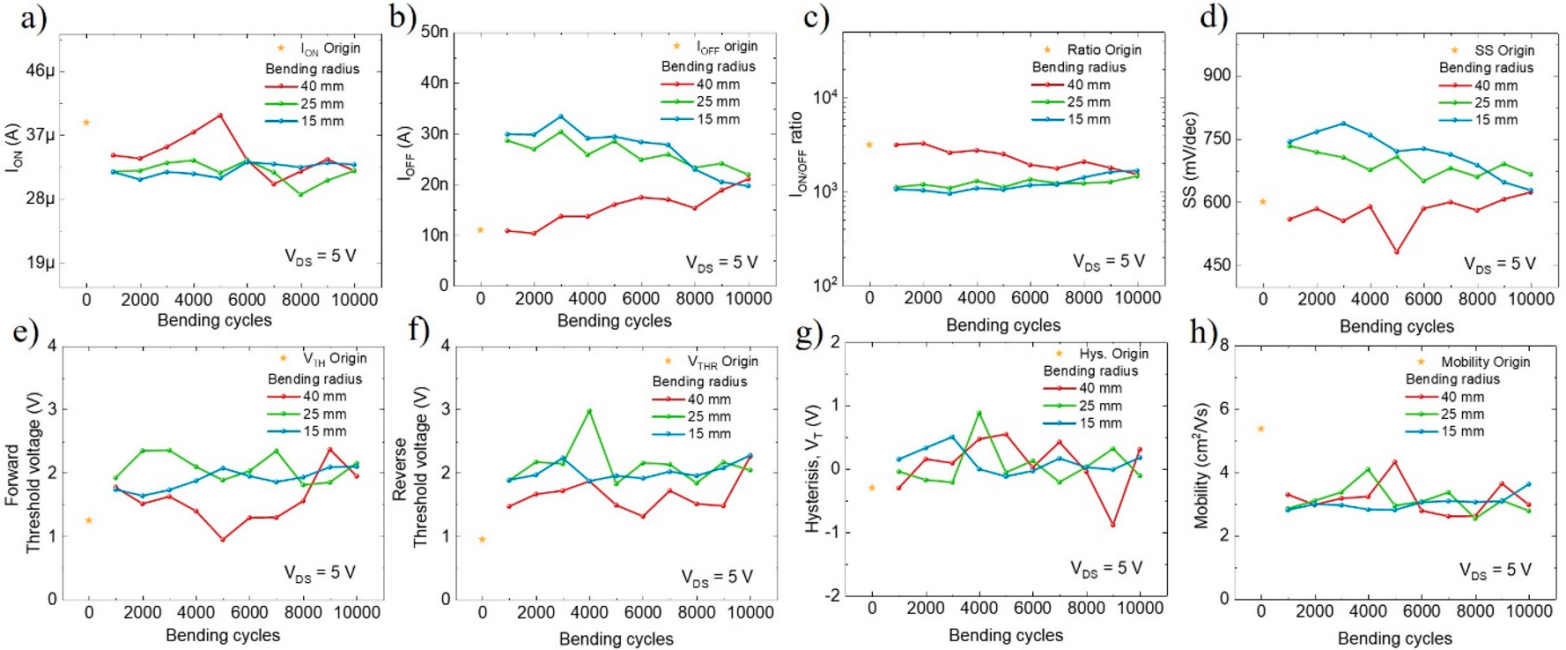
Extracted transistor parameters before (data shown for zero bending cycle) and after mechanical bending at various radii (from 40 mm to 25-, 15- and 5 mm). a) on-current, b) off-current, c) current on/off ratio, d) Subthreshold swing, e) threshold voltage (forward scan), f) threshold voltage, g) Hysteresis and h) mobility.

The performance degradation of devices was also observed under mechanical loadings, as flexible ICs need to remain stable, while under bending/twisting conditions. As shown in Figure 2i, the free-standing flexible printed transistors can be conformably placed on cylindrical objects such as Lab Vials etc. Static direct current (dc) measurements were carried out after 10000 bending cycles at different bending radius (ranging from 40 to 15 mm). The electrical characteristics after bending up to 10000 cycles (carried out after every 1000 cycles) at each bending radius are shown in Figure S3 (transfer curves) and Figure S4 (gate leakage). Like electric bias stability, the performed transfer scans were used to extract the key transistor performance metrics and shown in Figure 4. It is to note that the device performance metrics before bending is also displayed in the Figure 4 (data at zero bending cycle). Up to 7000–8000 bending cycles performed at 40 mm bending, the extracted data shows some variations. Such a small variation in extracted data could be attributed to the experimental errors arising from the placement of metal probes as measurement are performed after every 1000 bending cycles. Under ambient conditions, it is highly challenging to achieve exactly same interface quality between metal probe tips and the printed metal pads. However, after 8000 bending cycles at 40 mm radius, it is evident that the device showed small decrease in *I*_on_ (Figure 4a) and slight increase in *I*_off_ (Figure 4b). This leads to a small decrease in the *I*_on_/*I*_off_ ratio (Figure 4c) i.e., down from 3 × 10^3^ to ∼2 × 10^3^. After 8000 bending cycles, the increase in *I*_off_ could be due to the deterioration of SiN_*x*_ layer after bending induced stress. This can be further confirmed by the SS (Figure 4d) and hysteresis (Figure 4g) trend. There is increase in both SS and hysteresis after the device has undergone bending at 40 mm. Because of the deterioration of the dielectric gate, we assume that charges start to leak from gate electrodes onto trap sites (surface traps, interface traps, and dielectric traps). Further, after performing similar number of bending cycles at lower radius (25, and 15 mm), the *I*_on_ further decreased and the *I*_on_/*I*_off_ ratio reduced to ≈10^3^. The large reduction in device on-current after bending at lower bending radii (i.e., 25 mm and 15 mm) could be because of the strain experienced by the channel (Si NRs) as there was no further reduction in SS. The mechanical bending and the resulting strain are known to affect the semiconducting material’s band structure, which affects the effective mass and hence the mobility of the charge carriers.^70,71^ The change in the mobility has a direct effect over the source current of the transistor. The peak values of the device mobility after every 1000 cycles of bending were obtained for each bending radius (Figure 4h). It is evident from this set of data, the mobility decreased to around 3 cm^2^/(V s) (from 5 cm^2^/(V s) before bending). Nevertheless, the device operation was preserved despite the small decrease in the *I*_on_ and mobility.

Two of the straightforward approaches to minimize the impact of strain on flexible devices are to (i) minimize the substrate thickness and (ii) place the active electronic layers at neutral plane axis by using suitable encapsulation. Applying the first condition, taking the substrate thickness of ∼30 μm (including printed NS/epoxy), the strain at bending radii as low as 15 mm is minimized (∼0.1% at 15 mm bending).^72,73^ Further, the device stability was enhanced by exploring the second condition via printing a patterned encapsulation layer on top of the active channel and electrodes on the transistors. The deterioration of nanoscale device performance (large hysteresis in case of transistors) is expected when they are exposed to ambient environment mainly due to the infusion of humidity and/or adsorption of oxygen molecules.^61,63,64^ The solutions that have been explored to develop hysteresis-free devices include encapsulation and deposition of conformal dielectrics.^64^ The encapsulation layer can also enhance the mechanical endurance of the device by allowing the positioning of the most critical device layer in a neutral mechanical plane and also preventing the delamination of electronic materials. The delamination issue is more critical when inkjet printing is used to define the metal electrodes. The inkjet-based printed metals make heterogeneous contact interfaces and may experience the thermoelastic and mechanical stresses due to thermal mismatch,^74^ and elastic modulus mismatch respectively.^75^ While typical temperature conditions during device operation are low, high-temperature processing may be required during device fabrication, to cure the PI or to sinter the metallic Au ink (∼250 °C). We experienced the delamination of the printed metal pads for nonencapsulated devices when slight mechanical bending was applied (even when unintentionally applied while peeling off from the carrier glass substrate). As a result, we could not compare the nonencapsulated and encapsulated devices. Nevertheless, after encapsulation no delamination of metal pads was observed. Such a device configuration enables high flexibility along with stable electrical properties by bringing the devices at the neutral mechanical plane and prevents the device from experiencing any strain induced variations during cyclic bending. In the present case, the thickness of the printed dielectric is around 2–3 μm. Further increase in the thickness could possibly enhance the device stability and further reduce the variation of the output under bending conditions as shown in Figure 4.^76^

## 3 Conclusions

In summary, we have presented a printed route for development of high-performance n-and p-channel transistors on a flexible substrate. A roll-to-roll (R2R) compatible direct roll printing technique was employed to transfer silicon nanoribbons (Si NRs) and realize high mobility electronic layers from them. Instead of using the conventional metal deposition steps (photolithography, metal deposition and lift-off etc.), we used high resolution electrohydrodynamic based jet printing to deposit diffusive Au metal contacts for the first time on Si NRs. The scaled n-channel devices show high drain current and on/off ratio at small *V*_DS_ = 1 V. The investigated DC performance of transistors exhibits excellent electrical, thermal, and mechanical stability. Particularly, mobility of the devices remained stable after 10000 bending cycles at each bending radius of 40, 25, and 15 mm. In addition, the temperature-dependent dc measurements performed between 15 and 60 °C, demonstrate the stable device performance under extreme environments. Thus, the printed devices could provide a new route for the development of flexible, and robust complementary metal–oxide–semiconductor compatible printed logic circuits.

## 4 Methods

### 4.1 Silicon Nanoribbon Fabrication

The active channel of the printed transistors (i.e., Si NRs) are fabricated using a commercial silicon-on-insulator (SOI) wafer with a 70 nm thick Si (100) layer over 2 μm of buried oxide (B_OX_) purchased from the University Wafer, Inc. The as-received wafer first undergoes standard cleaning process (i.e., sequential ultrasonication in acetone, isopropyl alcohol (IPA), and deionized (DI) water for 5 min each). After cleaning, the lateral dimensions of NRs are defined following conventional photolithography and etching processes. First, S1805 photoresist is spun at 4000 rpm for 30 s followed by soft baking at 115 °C for 1 min. The resist is exposed to UV light through a photomask defining NRs array after developing in Microposit MF-319. Each NR is 55 μm in length and 5 μm in width (9NR in an array). The wafer is then laterally dry etched on the exposed regions by reactive ion etching (CHF_3_/O_2_, 50 sccm, 55 mTorr, 5 min). After the Si NR arrays are defined, the resist is removed, and 250 nm thick SiO_2_ is deposited using PECVD. A doping mask is patterned on the deposited oxide by dry etching, resulting in a 5 μm (undoped) channel length pattern on the NRs. The doping step is performed using spin-on dopants (SOD). n-Type doping is performed by diffusing phosphorus (Filmtronics, P451) at 1050 °C on the drain and source regions, whereas p-type doping is carried out by diffusing boron using (Filmtronics, P451) at 1050 °C. To achieve printable NRs arrays, the wafer is dipped on a buffered oxide etchant (BOE 5:1) to etch the mask and the underlying oxide. This results in suspension of the fabricated NRs anchored on the two extremities.

The nanoribbon (NR) structure has been selected here as it leads to higher the transfer yield, it is faster to underetch the NR (in width direction), and hence the overall process can be faster. The frame structure plays a critical role in defining the transfer yield particularly when it comes to transfer of nanoscale structures onto flexible substrates.^77^ The optimization study of the dependency of Si frame architectures on the transfer yield has been demonstrated in past works, where the resultant transfer yield was found to be the highest for structures with the frames that have densely spaced strips with 1:1 spacing ratio (NR width/NRs spacing).^77,78^ The aim of selecting multiple Si NRs connected in a single frame (single array) is to enhance the device on-current and reduce the device-to-device performance variation which is critical for realizing high performance transistors over large area.

### 4.2 Direct Roll Transfer Printing of Si NRs

The NRs are transfer-printed on to a flexible polyimide (PI) substrate using a custom-made direct roll setup. First, a layer of spin-on polyimide (PI-2545 from HD microsystems) is spun over a PI sheet of 25 μm thickness at 2000 rpm for 60 s. The spun PI is semicured at 120 °C for 2 min leading to a sticky adhesive layer. The prepared NR donor substrate is then placed to the roll printing setup (at the planar stage), and the PI substrate is placed on to the roller and brought into a direct physical contact with the donor substrate. This resulted into the detachment of NRs from the anchor points and lead to transfer on to the flexible PI substrate. The transfer process is completed by fully curing the spun PI at 250 °C for 2 h enhancing the adhesion of the NRs to the substrate.

### 4.3 Electrohydrodynamic (EHD) Printing

The PI substrate with printed NRs is gently attached on to a glass carrier substrate to have a flat and smooth surface. This is critical for EHD printing systems as the nozzle is brought in proximity of the substrate surface (<40 μm). Significant variation in the surface topography of the substrate will lead to variations in the nozzle–substrate separation distance and this, changes the effective electric field between the nozzle and substrate. This will have an impact on the amount of ejected ink and in an extreme case (±35 μm) the nozzle might touch the substrate and get broke. Before printing, a thermal treatment is performed at 120 °C to remove any remaining organic contaminant, followed by 1 min of plasma treatment inside (in situ) the high-resolution EHD printing system. The metal electrodes (drain/source/gate) were defined by printing five layers (passes) of commercial gold ink (CAu-2000 from ULVAC inc.). The ink is then cured at 250 °C for 2 h. The printing parameters are as follows: 75% sign wave, 300 V amplitude, 0 dc bias, 90 Hz frequency and stage speed of 0.5 mm/s. The device is finalised by printing the encapsulation layer based on a nanosilica/epoxy (NS/epoxy) dielectric ink from UTDots, Inc. The printing parameters used to print the dielectric ink are 50% sign wave, 250 V amplitude, 0 dc bias, 300 Hz frequency and stage speed of 0.75 mm/s. The encapsulation is cured at 150 °C for 30 min.

### 4.4 Electrical Characterization (Also Describing the Mechanical and Peltier System)

The electrical characterizations of the fabricated printed transistors were conducted using a Cascade Microtech Autoguard probe station connected to a semiconductor parameter analyzer (B1500A, Agilent) in dark conditions. The performance stability under wide temperature range (5–50 °C) was performed by placing the sample with devices on a Linkam PE120 Peltier system. The mechanical bending stress was applied on the sample using a commercial setup (Yuasa System DMLHP-TW).

## Supporting Information Available

Statistical data obtained for printed n-channel transistors from 5 NRFET devices: a) threshold voltage; b) Ion/off ratio, c) subthreshold swing; and d) peak field effect mobility. Statistical data obtained for printed p-channel transistors from 5 NRFET devices: a) threshold voltage; b) Ion/off ratio, c) subthreshold swing; and d) peak field effect mobility. Table S1 shows key performance metrics summarised for the fabricated flexible printed n- and pchannel transistor device. The statistical data (mean and standard deviation) is shown for each type of device. Transfer characteristics of the n-channel transistor performed after every 1000 bending cycles up to 10000 cycles: (a) bending at 40mm radius, (b) bending at 25mm radius, and (c) bending at 15mm radius. (PDF)

## References

[ref1] Song E.; Li J.; Won S. M.; Bai W.; Rogers J. A. Materials for Flexible Bioelectronic Systems as Chronic Neural Interfaces. Nat. Mater. 2020, 19 (6), 590–603. 10.1038/s41563-020-0679-7. 32461684

[ref2] Ling Y.; An T.; Yap L. W.; Zhu B.; Gong S.; Cheng W. Disruptive, Soft, Wearable Sensors. Adv. Mater. 2020, 32 (18), 1904664 10.1002/adma.201904664. 31721340

[ref3] Someya T.; Amagai M. Toward a New Generation of Smart Skins. Nat. Biotechnol. 2019, 37 (4), 382–388. 10.1038/s41587-019-0079-1. 30940942

[ref4] Dahiya R.; Yogeswaran N.; Liu F.; Manjakkal L.; Burdet E.; Hayward V.; Jörntell H. Large-Area Soft e-Skin: The Challenges Beyond Sensor Designs. Proc. IEEE 2019, 107 (10), 2016–2033. 10.1109/JPROC.2019.2941366.

[ref5] Dahiya R.; Akinwande D.; Chang J. S. Flexible Electronic Skin: From Humanoids to Humans [Scanning the Issue]. Proc. IEEE 2019, 107 (10), 2011–2015. 10.1109/JPROC.2019.2941665.

[ref6] Ozioko O.; Dahiya R. Smart Tactile Gloves for Haptic Interaction, Communication and Rehabilitation. Adv. Intell. Syst. 2022, 4 (2), 2100091 10.1002/aisy.202100091.

[ref7] Murali P. K.; Kaboli M.; Dahiya R. Intelligent In-Vehicle Interaction Technologies. Advanced Intelligent Systems 2022, 4 (2), 2100122 10.1002/aisy.202100122.

[ref8] Christou A.; Chirila R.; Dahiya R. Pseudo-Hologram with Aerohaptic Feedback for Interactive Volumetric Displays. Advanced Intelligent Systems 2022, 4 (2), 2100090 10.1002/aisy.202100090.

[ref9] Chen R. J.; Lu M. Y.; Chen T. Y.; Williamson D. F. K.; Mahmood F. Synthetic Data in Machine Learning for Medicine and Healthcare. Nature Biomedical Engineering 2021, 5 (6), 493–497. 10.1038/s41551-021-00751-8. PMC9353344 34131324

[ref10] Mullen E.; Morris M. A. Green Nanofabrication Opportunities in the Semiconductor Industry: A Life Cycle Perspective. Nanomaterials 2021, 11 (5), 1085 10.3390/nano11051085. 33922231 PMC8146645

[ref11] Dahiya A. S.; Christou A.; Neto J.; Zumeit A.; Shakthivel D.; Dahiya R. In Tandem Contact-Transfer Printing for High-Performance Transient Electronics. Advanced Electronic Materials 2022, 8 (9), 2200170 10.1002/aelm.202200170.

[ref12] Chakraborty M.; Kettle J.; Dahiya R. Electronic Waste Reduction Through Devices and Printed Circuit Boards Designed for Circularity. IEEE Journal on Flexible Electronics 2022, 1 (1), 4–23. 10.1109/JFLEX.2022.3159258.

[ref13] Dahiya A. S.; Shakthivel D.; Kumaresan Y.; Zumeit A.; Christou A.; Dahiya R. High-performance Printed Electronics Based on Inorganic Semiconducting Nano to Chip Scale Structures. Nano Convergence 2020, 7 (1), 33 10.1186/s40580-020-00243-6. 33034776 PMC7547062

[ref14] Khan S.; Lorenzelli L.; Dahiya R. S. Technologies for Printing Sensors and Electronics Over Large Flexible Substrates: A Review. IEEE Sensors Journal 2015, 15 (6), 3164–3185. 10.1109/JSEN.2014.2375203.

[ref15] Chakraborty M.; Nikbakhtnasrabadi F.; Dahiya R. Hybrid Integration of Screen-Printed RFID Tags and Rigid Microchip on Paper. IEEE Journal on Flexible Electronics 2022, 1 (2), 107–113. 10.1109/JFLEX.2022.3165515.

[ref16] Kumaresan Y.; Kim H.; Pak Y.; Poola P. K.; Lee R.; Lim N.; Ko H. C.; Jung G. Y.; Dahiya R. Omnidirectional Stretchable Inorganic-Material-Based Electronics with Enhanced Performance. Adv. Electron Mater. 2020, 6 (7), 2000058 10.1002/aelm.202000058.

[ref17] Sun J.; Sapkota A.; Park H.; Wesley P.; Jung Y.; Maskey B. B.; Kim Y.; Majima Y.; Ding J.; Ouyang J.; Guo C.; Lefebvre J.; Li Z.; Malenfant P. R. L.; Javey A.; Cho G. Fully R2R-Printed Carbon-Nanotube-Based Limitless Length of Flexible Active-Matrix for Electrophoretic Display Application. Advanced Electronic Materials 2020, 6 (4), 1901431 10.1002/aelm.201901431.

[ref18] Chung S.; Cho K.; Lee T. Recent Progress in Inkjet-Printed Thin-Film Transistors. Advanced Science 2019, 6 (6), 1801445 10.1002/advs.201801445. 30937255 PMC6425446

[ref19] Klauk H. Organic Electronics: Materials, Manufacturing and Applications; John Wiley & Sons, 2006; p 422–428.

[ref20] Bonnassieux Y.; Brabec C. J.; Cao Y.; Carmichael T. B.; Chabinyc M. L.; Cheng K.-T.; Cho G.; Chung A.; Cobb C. L.; Distler A.; Egelhaaf H.-J.; Grau G.; Guo X.; Haghiashtiani G.; Huang T.-C.; Hussain M. M.; Iniguez B.; Lee T.-M.; Li L.; Ma Y.; Ma D.; McAlpine M. C.; Ng T. N.; Österbacka R.; Patel S. N.; Peng J.; Peng H.; Rivnay J.; Shao L.; Steingart D.; Street R. A.; Subramanian V.; Torsi L.; Wu Y. The 2021 Flexible and Printed Electronics Roadmap. Flexible and Printed Electronics 2021, 6 (2), 023001 10.1088/2058-8585/abf986.

[ref21] Beniwal A.; Ganguly P.; Aliyana A. K.; Khandelwal G.; Dahiya R. Screen-printed graphene-carbon ink based disposable humidity sensor with wireless communication. Sens. Actuators, B 2023, 374, 132731 10.1016/j.snb.2022.132731.

[ref22] Bhattacharjee M.; Nikbakhtnasrabadi F.; Dahiya R. Printed Chipless Antenna as Flexible Temperature Sensor. IEEE Internet of Things Journal 2021, 8 (6), 5101–5110. 10.1109/JIOT.2021.3051467.

[ref23] Suganuma K. Introduction to Printed Electronics. Springer: 2014.

[ref24] Dahiya A. S.; Zumeit A.; Christou A.; Dahiya R. High-Performance n-Channel Printed Transistors on Biodegradable Substrate for Transient Electronics. Advanced Electronic Materials 2022, 8, 2200098 10.1002/aelm.202200098.

[ref25] García Núñez C.; Liu F.; Xu S.; Dahiya R. Integration Techniques for Micro/Nanostructure-based Large-Area Electronics; Cambridge University Press: Cambridge, 2018.

[ref26] Neto J.; Chirila R.; Dahiya A. S.; Christou A.; Shakthivel D.; Dahiya R. Skin-Inspired Thermoreceptors-Based Electronic Skin for Biomimicking Thermal Pain Reflexes. Advanced Science 2022, 9, 2201525 10.1002/advs.202201525. 35876394 PMC9507360

[ref27] Paul A.; Yogeswaran N.; Dahiya R. Ultra-Flexible Biodegradable Pressure Sensitive Field Effect Transistors for Hands-Free Control of Robot Movements. Advanced Intelligent Systems 2022, 4, 2200183 10.1002/aisy.202200183.

[ref28] Liu F.; Deswal S.; Christou A.; Shojaei Baghini M.; Chirila R.; Shakthivel D.; Chakraborty M.; Dahiya R. Printed Synaptic Transistor-Based Electronic Skin for Robots to Feel and Learn. Sci. Robot. 2022, 7 (67), eabl7286 10.1126/scirobotics.abl7286. 35648845

[ref29] Godard N.; Allirol L.; Latour A.; Glinsek S.; Gérard M.; Polesel J.; Domingues Dos Santos F.; Defay E. 1-mW Vibration Energy Harvester Based on a Cantilever with Printed Polymer Multilayers. Cell Reports Physical Science 2020, 1 (6), 100068 10.1016/j.xcrp.2020.100068.

[ref30] Sim K.; Chen S.; Li Z.; Rao Z.; Liu J.; Lu Y.; Jang S.; Ershad F.; Chen J.; Xiao J.; Yu C. Three-dimensional Curvy Electronics Created Using Conformal Additive Stamp Printing. Nature Electronics 2019, 2 (10), 471–479. 10.1038/s41928-019-0304-4.

[ref31] Khandelwal G.; Dahiya R. Self-Powered Active Sensing Based on Triboelectric Generators. Adv. Mater. 2022, 34 (33), 2200724 10.1002/adma.202200724. 35445458

[ref32] Nikbakhtnasrabadi F.; Hosseini E. S.; Dervin S.; Shakthivel D.; Dahiya R. Smart Bandage with Inductor-Capacitor Resonant Tank Based Printed Wireless Pressure Sensor on Electrospun Poly-L-Lactide Nanofibers. Advanced Electronic Materials 2022, 8 (7), 2101348 10.1002/aelm.202101348.

[ref33] Ma S.; Kumaresan Y.; Dahiya A. S.; Dahiya R. Ultra-Thin Chips with Printed Interconnects on Flexible Foils. Advanced Electronic Materials 2022, 8, 2101029 10.1002/aelm.202101029.

[ref34] Liu F.; Deswal S.; Christou A.; Sandamirskaya Y.; Kaboli M.; Dahiya R. Neuro-inspired Electronic Skin for Robots. Sci. Robot. 2022, 7 (67), eabl7344 10.1126/scirobotics.abl7344. 35675450

[ref35] Lau P. H.; Takei K.; Wang C.; Ju Y.; Kim J.; Yu Z.; Takahashi T.; Cho G.; Javey A. Fully Printed, High Performance Carbon Nanotube Thin-Film Transistors on Flexible Substrates. Nano Lett. 2013, 13 (8), 3864–3869. 10.1021/nl401934a. 23899052

[ref36] Myny K. The Development of Flexible Integrated Circuits Based on Thin-Film Transistors. Nature Electronics 2018, 1 (1), 30–39. 10.1038/s41928-017-0008-6.

[ref37] Biggs J.; Myers J.; Kufel J.; Ozer E.; Craske S.; Sou A.; Ramsdale C.; Williamson K.; Price R.; White S. A Natively Flexible 32-bit Arm Microprocessor. Nature 2021, 595 (7868), 532–536. 10.1038/s41586-021-03625-w. 34290427

[ref38] Myny K.; van Veenendaal E.; Gelinck G. H.; Genoe J.; Dehaene W.; Heremans P. An 8-Bit, 40-Instructions-Per-Second Organic Microprocessor on Plastic Foil. IEEE J. Solid-State Circuits 2012, 47 (1), 284–291. 10.1109/JSSC.2011.2170635.

[ref39] Myny K.; Smout S.; Rockelé M.; Bhoolokam A.; Ke T. H.; Steudel S.; Obata K.; Marinkovic M.; Pham D. V.; Hoppe A. 30.1 8b Thin-film Microprocessor Using a Hybrid Oxide-Organic Complementary Technology with Inkjet-Printed PROM Memory. IEEE Int. Solid-State Circuits Conf. Digest Tech. Pap. 2014, 486–487. 10.3390/mi13040509.

[ref40] Liu K.; Ouyang B.; Guo X.; Guo Y.; Liu Y. Advances in Flexible Organic Field-Effect Transistors and Their Applications for Flexible Electronics. npj Flex. Electron. 2022, 6 (1), 1 10.1038/s41528-022-00133-3.

[ref41] Ozer E.; Kufel J.; Myers J.; Biggs J.; Brown G.; Rana A.; Sou A.; Ramsdale C.; White S. A Hardwired Machine Learning Processing Engine Fabricated with Submicron Metal-Oxide Thin-Film Transistors on a Flexible Substrate. Nat. Electron 2020, 3 (7), 419–425. 10.1038/s41928-020-0437-5.

[ref42] Liu F.; Dahiya A. S.; Dahiya R. A Flexible Chip with Embedded Intelligence. Nat. Electron 2020, 3 (7), 358–359. 10.1038/s41928-020-0446-4.

[ref43] Gupta S.; Navaraj W. T.; Lorenzelli L.; Dahiya R. Ultra-Thin Chips for High-Performance Flexible Electronics. npj Flex. Electron. 2018, 2 (1), 8 10.1038/s41528-018-0021-5.

[ref44] Ma S.; Kumaresan Y.; Dahiya A. S.; Lorenzelli L.; Dahiya R. Flexible Tactile Sensors using AlN and MOSFETs based Ultra-thin Chips. IEEE Sensors J. 2022, 3140651 10.1109/JSEN.2022.3140651.

[ref45] Zumeit A.; Dahiya A. S.; Christou A.; Mukherjee R.; Dahiya R. Printed GaAs Microstructures-Based Flexible High-Performance Broadband Photodetectors. Advanced Materials Technologies 2022, 7, 2200772 10.1002/admt.202200772.

[ref46] Zumeit A.; Dahiya A. S.; Christou A.; Shakthivel D.; Dahiya R. Direct Roll Transfer Printed Silicon Nanoribbon Arrays based High-Performance Flexible Electronics. npj Flex. Electron. 2021, 5, 18 10.1038/s41528-021-00116-w.

[ref47] Zumeit A.; Dahiya A. S.; Christou A.; Dahiya R. High performance P-Channel Transistors on Flexible Substrate Using Direct Roll Transfer Stamping. Jpn. J. Appl. Phys. 2022, 61, SC1042 10.35848/1347-4065/ac40ab.

[ref48] Lei Q.; He J.; Li D. Electrohydrodynamic 3D printing of Layer-Specifically Oriented, Multiscale Conductive Scaffolds for Cardiac Tissue Engineering. Nanoscale 2019, 11 (32), 15195–15205. 10.1039/C9NR04989D. 31380883

[ref49] Galliker P.; Schneider J.; Eghlidi H.; Kress S.; Sandoghdar V.; Poulikakos D. Direct Printing of Nanostructures by Electrostatic Autofocussing of Ink Nanodroplets. Nat. Commun. 2012, 3 (1), 890 10.1038/ncomms1891. 22692533

[ref50] Park J.-U.; Hardy M.; Kang S. J.; Barton K.; Adair K.; Mukhopadhyay D. k.; Lee C. Y.; Strano M. S.; Alleyne A. G.; Georgiadis J. G.; Ferreira P. M.; Rogers J. A. High-resolution Electrohydrodynamic Jet Printing. Nat. Mater. 2007, 6 (10), 782–789. 10.1038/nmat1974. 17676047

[ref51] Xu Q.; Zhao J.; Pecunia V.; Xu W.; Zhou C.; Dou J.; Gu W.; Lin J.; Mo L.; Zhao Y.; Cui Z. Selective Conversion from p-Type to n-Type of Printed Bottom-Gate Carbon Nanotube Thin-Film Transistors and Application in Complementary Metal–Oxide–Semiconductor Inverters. ACS Appl. Mater. Interfaces 2017, 9 (14), 12750–12758. 10.1021/acsami.7b01666. 28337913

[ref52] Xu W.; Dou J.; Zhao J.; Tan H.; Ye J.; Tange M.; Gao W.; Xu W.; Zhang X.; Guo W.; Ma C.; Okazaki T.; Zhang K.; Cui Z. Printed Thin Film Transistors and CMOS Inverters Based on Semiconducting Carbon Nanotube Ink Purified by a Nonlinear Conjugated Copolymer. Nanoscale 2016, 8 (8), 4588–4598. 10.1039/C6NR00015K. 26847814

[ref53] Cao C.; Andrews J. B.; Kumar A.; Franklin A. D. Improving Contact Interfaces in Fully Printed Carbon Nanotube Thin-Film Transistors. ACS Nano 2016, 10 (5), 5221–5229. 10.1021/acsnano.6b00877. 27097302

[ref54] Su Y.; Du J.; Sun D.; Liu C.; Cheng H. Reduced Graphene Oxide With a Highly Restored π-Conjugated Structure for Inkjet Printing and its Use in All-Carbon Transistors. Nano Research 2013, 6 (11), 842–852. 10.1007/s12274-013-0362-2.

[ref55] Su Y.; Jia S.; Du J.; Yuan J.; Liu C.; Ren W.; Cheng H. Direct Writing of Graphene Patterns and Devices on Graphene Oxide Films by Inkjet Reduction. Nano Research 2015, 8 (12), 3954–3962. 10.1007/s12274-015-0897-5.

[ref56] Mondal S. K.; Biswas A.; Pradhan J. R.; Dasgupta S. Inkjet-Printed MoS2 Transistors with Predominantly Intraflake Transport. Small Methods 2021, 5 (12), 2100634 10.1002/smtd.202100634. 34928044

[ref57] Lin Z.; Liu Y.; Halim U.; Ding M.; Liu Y.; Wang Y.; Jia C.; Chen P.; Duan X.; Wang C.; Song F.; Li M.; Wan C.; Huang Y.; Duan X. Solution-Processable 2D Semiconductors for High-Performance Large-Area Electronics. Nature 2018, 562 (7726), 254–258. 10.1038/s41586-018-0574-4. 30283139

[ref58] Liang K.; Ren H.; Li D.; Wang Y.; Tang Y.; Zhao M.; Wang H.; Li W.; Zhu B. Fully-Printed Flexible n-Type Tin Oxide Thin-Film Transistors and Logic Circuits. Journal of Materials Chemistry C 2021, 9 (35), 11662–11668. 10.1039/D1TC01512E.

[ref59] Zumeit A.; Navaraj W. T.; Shakthivel D.; Dahiya R. Nanoribbon-Based Flexible High-Performance Transistors Fabricated at Room Temperature. Adv. Electron Mater. 2020, 6 (4), 1901023 10.1002/aelm.201901023.

[ref60] Dahiya A. S.; Sporea R. A.; Poulin-Vittrant G.; Alquier D. Stability evaluation of ZnO nanosheet based source-gated transistors. Sci. Rep. 2019, 9 (1), 2979 10.1038/s41598-019-39833-8. 30814622 PMC6393496

[ref61] Dahiya A. S.; Opoku C.; Poulin-Vittrant G.; Camara N.; Daumont C.; Barbagiovanni E. G.; Franzò G.; Mirabella S.; Alquier D. Flexible Organic/Inorganic Hybrid Field-Effect Transistors with High Performance and Operational Stability. ACS Appl. Mater. Interfaces 2017, 9 (1), 573–584. 10.1021/acsami.6b13472. 28001361

[ref62] Cheng Z.; Pang C.-S.; Wang P.; Le S. T.; Wu Y.; Shahrjerdi D.; Radu I.; Lemme M. C.; Peng L.-M.; Duan X.; Chen Z.; Appenzeller J.; Koester S. J.; Pop E.; Franklin A. D.; Richter C. A. How to Report and Benchmark Emerging Field-Effect Transistors. Nature Electronics 2022, 5 (7), 416–423. 10.1038/s41928-022-00798-8.

[ref63] Dahiya A. S.; Opoku C.; Oshman C.; Poulin-Vittrant G.; Cayrel F.; Hue L.-P. T. H.; Alquier D.; Camara N. Zinc Oxide Sheet Field-Effect Transistors. Appl. Phys. Lett. 2015, 107 (3), 033105 10.1063/1.4927270.

[ref64] Lu Y. X.; Lin C. T.; Tsai M. H.; Lin K. C. Review-Hysteresis in Carbon Nano-Structure Field Effect Transistor. Micromachines 2022, 13 (4), 509 10.3390/mi13040509. 35457813 PMC9029578

[ref65] Zschieschang U.; Borchert J. W.; Giorgio M.; Caironi M.; Letzkus F.; Burghartz J. N.; Waizmann U.; Weis J.; Ludwigs S.; Klauk H. Roadmap to Gigahertz Organic Transistors. Adv. Funct Mater. 2020, 30 (20), 1903812 10.1002/adfm.201903812.

[ref66] Shin D.; Bae M. S.; Yun I. Instability of Oxide Thin Film Transistor Under Electrical–Mechanical Hybrid Stress for Foldable Display. Microelectronics Reliability 2016, 64, 109–112. 10.1016/j.microrel.2016.07.017.

[ref67] Jeong J. K.; Won Yang H.; Jeong J. H.; Mo Y.-G.; Kim H. D. Origin of Threshold Voltage Instability in Indium-Gallium-Zinc Oxide Thin Film Transistors. Appl. Phys. Lett. 2008, 93 (12), 123508 10.1063/1.2990657.

[ref68] Dahiya A. S.; Opoku C.; Sporea R. A.; Sarvankumar B.; Poulin-Vittrant G.; Cayrel F.; Camara N.; Alquier D. Single-Crystalline ZnO Sheet Source-Gated Transistors. Sci. Rep. 2016, 6 (1), 19232 10.1038/srep19232. 26757945 PMC4725757

[ref69] Dahiya A. S.; Opoku C.; Cayrel F.; Valente D.; Poulin-Vittrant G.; Camara N.; Alquier D. Temperature Dependence of Charge Transport in Zinc Oxide Nanosheet Source-Gated Transistors. Thin Solid Films 2016, 617, 114–119. 10.1016/j.tsf.2016.02.021.

[ref70] Vilouras A.; Heidari H.; Gupta S.; Dahiya R. Modeling of CMOS Devices and Circuits on Flexible Ultrathin Chips. IEEE Trans. Electron Devices 2017, 64 (5), 2038–2046. 10.1109/TED.2017.2668899.

[ref71] Gupta S.; Heidari H.; Vilouras A.; Lorenzelli L.; Dahiya R. Device Modelling for Bendable Piezoelectric FET-Based Touch Sensing System. IEEE Transactions on Circuits and Systems I: Regular Papers 2016, 63 (12), 2200–2208. 10.1109/TCSI.2016.2615108.

[ref72] Daus A.; Vaziri S.; Chen V.; Köroğlu Ç.; Grady R. W.; Bailey C. S.; Lee H. R.; Schauble K.; Brenner K.; Pop E. High-Performance Flexible Nanoscale Transistors Based on Transition Metal Dichalcogenides. Nature Electronics 2021, 4 (7), 495–501. 10.1038/s41928-021-00598-6.

[ref73] Lewis J. Material Challenge for Flexible Organic Devices. Mater. Today 2006, 9 (4), 38–45. 10.1016/S1369-7021(06)71446-8.

[ref74] Suhir E. An Approximate Analysis of Stresses in Multilayered Elastic Thin Films. Journal of Applied Mechanics 1988, 55 (1), 143–148. 10.1115/1.3173620.

[ref75] Naserifar N.; LeDuc P. R.; Fedder G. K. Material Gradients in Stretchable Substrates toward Integrated Electronic Functionality. Adv. Mater. 2016, 28 (18), 3584–3591. 10.1002/adma.201505818. 26989814

[ref76] Kim D.-H.; Ahn J.-H.; Choi W. M.; Kim H.-S.; Kim T.-H.; Song J.; Huang Y. Y.; Liu Z.; Lu C.; Rogers J. A. Stretchable and Foldable Silicon Integrated Circuits. Science 2008, 320 (5875), 507–511. 10.1126/science.1154367. 18369106

[ref77] Grierson D.; Flack F.; Lagally M.; Turner K. Rolling-Based Direct-Transfer Printing: A process for Large-area transfer of Micro and Nanostructures onto Flexible Substrates. J. Appl. Phys. 2016, 120 (9), 093103 10.1063/1.4961407.

[ref78] Sun L.; Qin G.; Seo J. H.; Celler G. K.; Zhou W.; Ma Z. 12-GHz Thin-Film Transistors on Transferrable Silicon Nanomembranes for High-Performance Flexible Electronics. Small 2010, 6 (22), 2553–2557. 10.1002/smll.201000522. 20878631

