# Supplementary material for: Printed n- and p-Channel Transistors using Silicon Nanoribbons Enduring Electrical, Thermal, and Mechanical Stress
Source: ACS Appl Mater Interfaces. 2023 Feb 12;15(7):9618–28. doi: 10.1021/acsami.2c20569 (PMC9990968; doi:10.1021/acsami.2c20569)
Supplement: Supplementary file 1 — am2c20569_si_001.pdf [file am2c20569_si_001.pdf]

Supporting Information

Printed n- and p-channel Transistors using Silicon Nanoribbons Enduring Electrical, Thermal, and Mechanical Stress

João Neto[1], Abhishek Singh Dahiya[1], Ayoub Zumeit[1], Adamos Christou[1], Sihang Ma[1], and Ravinder Dahiya[2]*

[1]James Watt School of Engineering, University of Glasgow, Glasgow G12 8QQ, U.K

[2]Bendable Electronics and Sustainable Technologies (BEST) Group, Electrical and Computer Engineering Department, Northeastern University, Boston, MA 02115, USA

*Corresponding author: Prof. Ravinder Dahiya

E-mail: r.dahiya@northeastern.edu

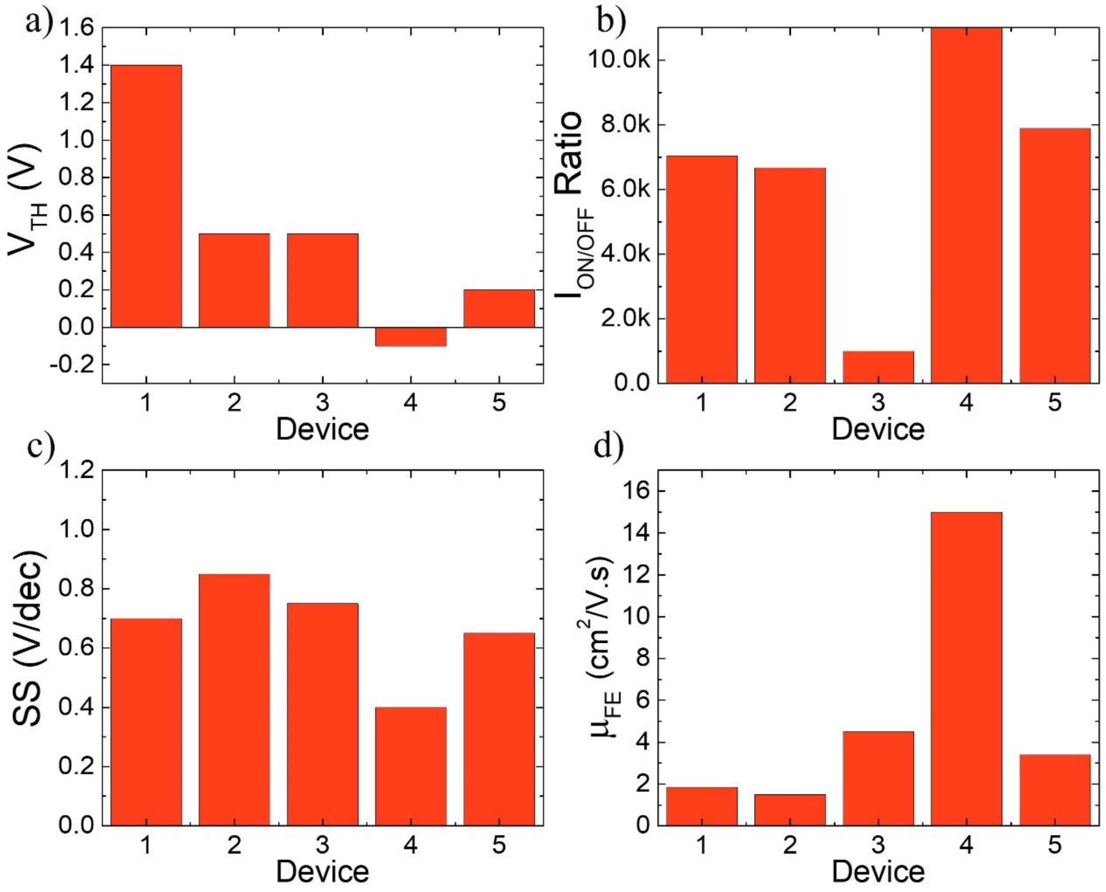

Figure S1. Statistical data obtained for printed n-channel transistors from 5 NRFET devices: a) threshold voltage; b) $I_{on/off}$ ratio, c) subthreshold swing; and d) peak field effect mobility.

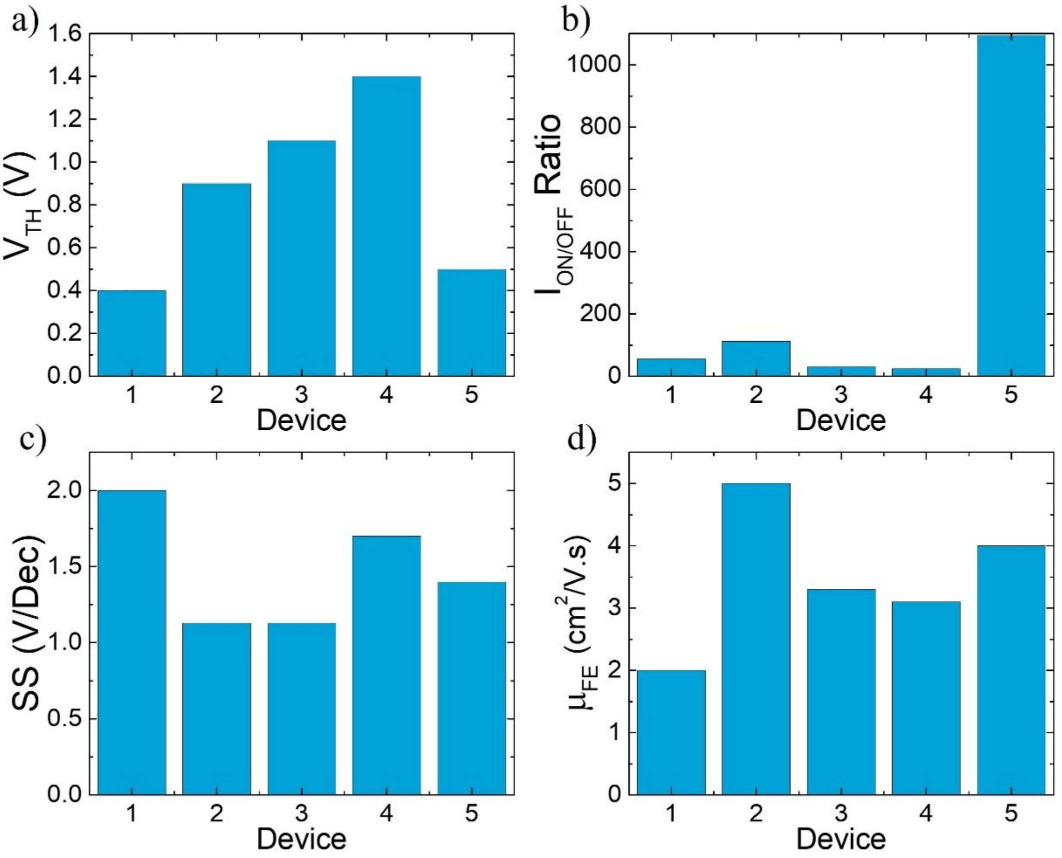

Figure S2. Statistical data obtained for printed p-channel transistors from 5 NRFET devices: a) threshold voltage; b) $I_{on/off}$ ratio, c) subthreshold swing; and d) peak field effect mobility.

Table S1. Key performance metrics summarised for the fabricated flexible printed n- and p-channel transistor device. The statistical data (mean and standard deviation) is shown for each type of device.

**Channel-type**	**$I_{on/off}$ ratio**	**μ_{eff} (cm^2/ Vs)**	**S-S (V/dec)**	**V_T (V)**
**N-type**	9000 ± 8000	5.3 ± 5.6	0.67 ± 0.16	0.5 ± 0.56
**P-type**	263 ± 466	3.5 ± 1.1	1.4 ± 0.37	0.86 ± 0.4

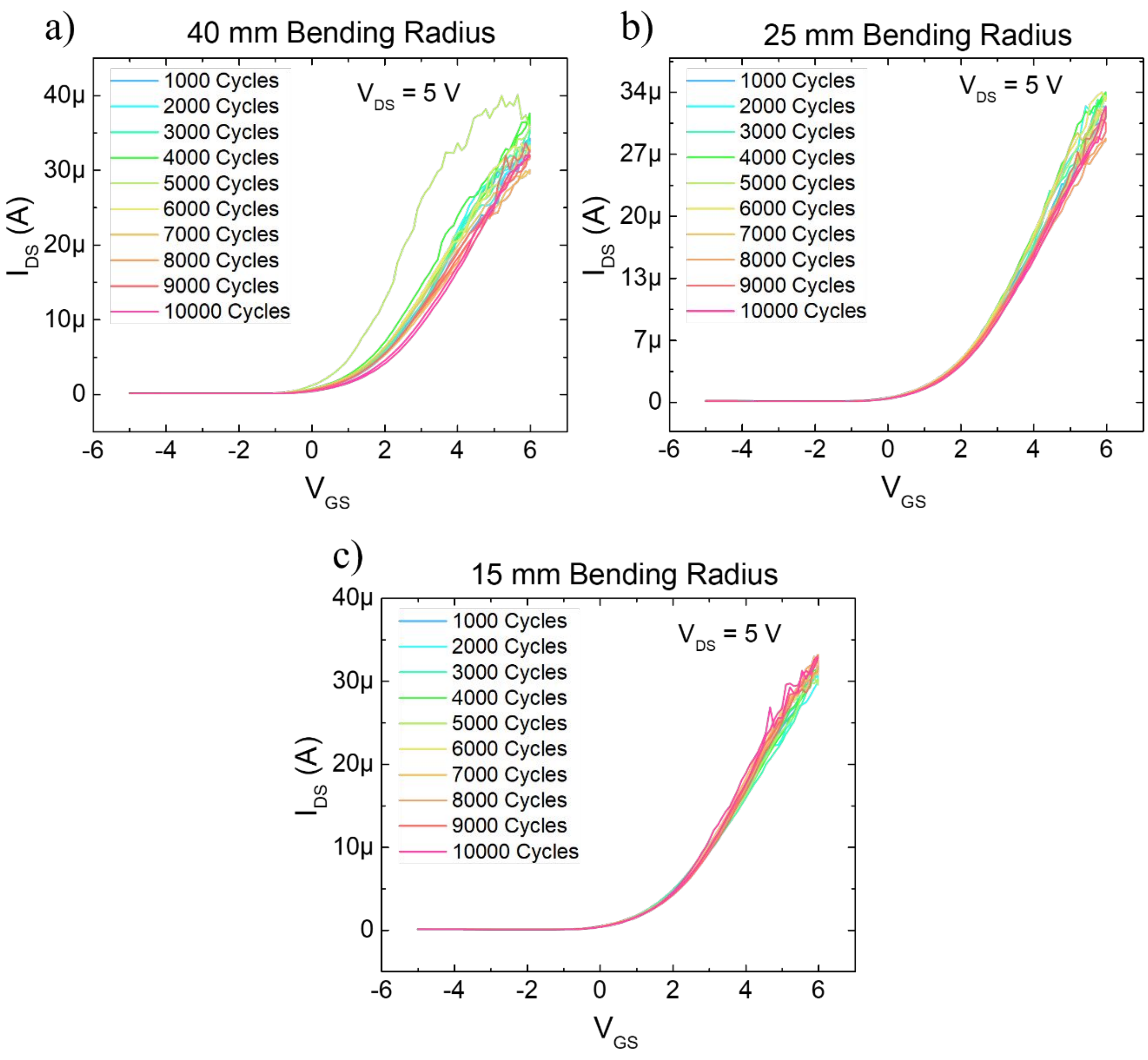

Figure S3. Transfer characteristics of the n-channel transistor performed after every 1000 bending cycles up to 10000 cycles: (a) bending at 40mm radius, (b) bending at 25mm radius, and (c) bending at 15mm radius.

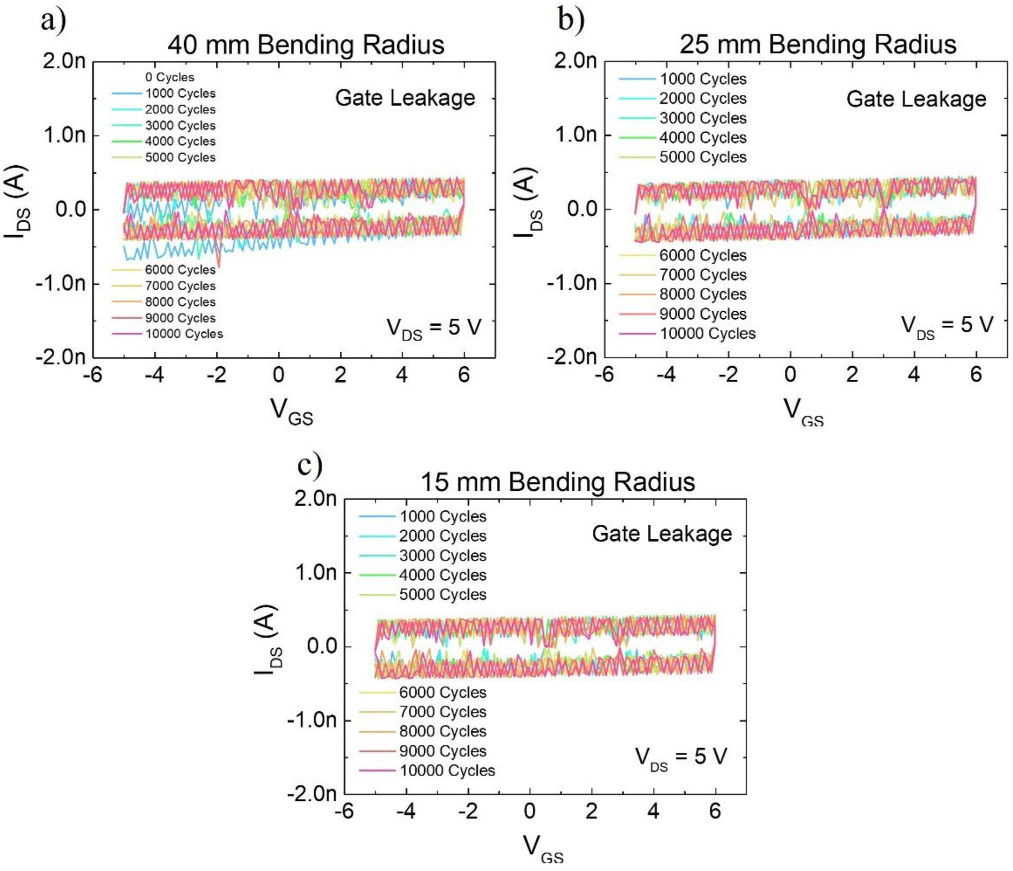

Figure S4. Gate leakage current for the n-channel transistor performed after every 1000 bending cycles up to 10000 cycles: (a) bending at 40mm radius, (b) bending at 25mm radius, and (c) bending at 15mm radius.
